# Factors Influencing Self-Efficacy and Self-Management among Patients with Pre-End-Stage Renal Disease (Pre-ESRD)

**DOI:** 10.3390/healthcare9030266

**Published:** 2021-03-02

**Authors:** Pao-Chin Lai, Shu-Fang Vivienne Wu, Javad Alizargar, Satriya Pranata, Juin-Ming Tsai, Nan-Chen Hsieh

**Affiliations:** 1 Department of Nursing, Shin Kong Wu Ho-Su Memorial Hospital, Taipei 111, Taiwan; emily500903@yahoo.com.tw; 2 School of Nursing, National Taipei University of Nursing and Health Sciences, Taipei 112, Taiwan; satriya.pranata@unimus.ac.id; 3 Research Center for Healthcare Industry Innovation, National Taipei University of Nursing and Health Sciences, Taipei 112, Taiwan; 4 Faculty of Nursing and Health Sciences, Muhammadiyah University of Semarang, Jawa Tengah 50273, Indonesia; 5 Department of Gerontological Health Care, National Taipei University of Nursing and Health Sciences, Taipei 112, Taiwan; juinming@ntunhs.edu.tw; 6 Department of Information Management, National Taipei University of Nursing and Health Sciences, Taipei 112, Taiwan; nchsieh@ntunhs.edu.tw

**Keywords:** anxiety, chronic kidney disease patients, depression, end-stage renal disease (ESRD), self-efficacy, self-management

## Abstract

Aim: Chronic kidney disease (CKD) is an emerging major public health issue that leads to end-stage kidney disease (ESRD). Factors influencing the self-management and self-efficacy of ESRD patients are still under investigation. The objective of this study is to evaluate the association of depression and anxiety with self-management and self-efficacy in patients with pre-ESRD. Methods: Patients in the department of nephrology of a regional hospital in Taiwan were invited to participate and were included in our study if they had a confirmed diagnosis of early-stage CKD, were more than 20 years old, and could converse in Mandarin Chinese or Taiwanese. Patients diagnosed with depression, who could not execute self-care, or who had cognitive deficits were excluded. In total, this cross-sectional study included 112 pre-ESRD patients. We used the Chinese versions of the hospital anxiety and depression scale (HADS), the chronic kidney disease self-efficacy instrument (CKD-SE), and the chronic kidney disease self-management instrument (CKD-SM) as the questionnaire. Spearman’s rank correlation and logistic regressions were used to analyze the data. Results: The top quartile of self-management and self-efficacy scores (28 patients) was defined as high self-management and -efficacy, respectively, and the lower three quartiles as low self-management and -efficacy. The logistic regression analysis showed that having depression decreased the odds of having high self-management by 75.4% and high self-efficacy by 75.1%. Having an education level of senior high school or above increased the odds ratios for having high self-management and high self-efficacy to 4.47 and 3.56 (all *p*-values < 0.05). Conclusion: Controlling depression as well as increasing the level of education can potentially increase self-management and self-efficacy in pre-ESRD patients.

## 1. Background 

Chronic kidney disease (CKD) is an important and prevalent disease that is emerging as a major public health issue. The global prevalence of CKD as of 2017 was 9.1% (697.5 million cases), causing 1.2 million deaths and ranking as the twelfth leading cause of death worldwide [1]. CKD places a large burden on the medical system since it leads to end-stage renal disease (ESRD), which can significantly reduce the quality of life and is associated with a high mortality rate. Taiwan has a high prevalence of ESRD, and this condition requires greater attention than it is currently receiving [2]. 

Common treatment options for ESRD include maintenance haemodialysis, regular attendance at a dialysis center, adhering to fluid-intake protocols, medications, and lifestyle changes. Poor adherence to these treatments and lifestyle changes results in an increase in mortality and morbidity. The psychological and social stress associated with the condition influence survival rate and reduce quality of life [3]. Psychiatric disorders commonly co-exist with CKD and ESRD, and this may explain the high prevalence of depression and anxiety in ESRD patients [4]. In addition to physical and nutritional impairment, old age, and heart failure, depression is also considered a major risk factor for mortality in these patients [5].

Curtin and Mapes define self-management in ESRD patients as positive efforts that they make to monitor and control their own symptoms, perform their own healthcare, and make use of available medical resources, among other actions, so as to further reduce the occurrence of comorbidities and enable them to live their preferred lifestyle [6]. Self-management can improve anxiety, depression, and quality of life [3,7,8]. Poorer self-management in patients with ESRD has been shown to be correlated with higher mortality. A stronger self-management also decreases the complications of ESRD [9]. The literature shows that self-management in such patients is not currently ideal, but the influence of various factors on self-management has not yet been studied in detail [3]. However, education and knowledge, in addition to depression and anxiety, have been found to affect self-management in these patients [10]. By studying these related factors, we can suggest interventions to improve their self-management. 

Self-efficacy was found to be an independent predictor of self-management in ESRD patients [10]. Self-efficacy is defined as a patient’s confidence in their ability to adhere to the treatment and manage their disease [11]. Self-efficacy affects the amount of effort patients put into their actions to deal with their disease. It also influences how well they react when facing obstacles and failures, as well as the strength of their resilience when facing adverse situations caused by their medical condition and disease comorbidities [12,13,14,15]. Self-efficacy helps determine how long patients will be persistent when facing obstacles caused by their disease [14]. Low self-efficacy usually increases problems and worsens disease-related conditions. Patients with low self-efficacy have a greater chance of developing emotional and social problems, including mental-health conditions such as anxiety and depression [16,17]. Self-efficacy and self-care are significant predictors of quality of life and depression among patients receiving haemodialysis [18]. Self-efficacy is correlated to self-management in ESRD patients, but the factors influencing this relationship are still under investigation [3]. Therefore, the objective of this study is to evaluate the association of various factors with self-management and self-efficacy in patients with pre-ESRD.

## 2. Methods

### 2.1. Study Design

A cross-sectional design was used. Purposive sampling was used to recruit subjects from the department of nephrology of a regional hospital in Taiwan. 

### 2.2. Subjects and Setting

This study focused on pre-ESRD patients who had been referred to the abovementioned department of nephrology. The criteria for subject selection were a confirmed diagnosis in the patient’s medical record of early-stage CKD by a physician, an age of more than 20 years, and the ability to converse in Mandarin Chinese or Taiwanese. The exclusion criteria were a diagnosis of depression, the inability to execute self-care (e.g., visually impaired in both eyes), and the presence of cognitive deficits (such as dementia, this was based on the patient’s medical record). We identified 112 patients who met the inclusion criteria and were willing to participate in this study. Then, we explained the study to the participants and had them sign the consent form and complete the survey. The basic demographic data and survey results were obtained during a single session. 

### 2.3. Outcome Measures

Demographic characteristics such as age, CKD stage, time since CKD diagnosis, educational level, sex, marital status, occupation, religion, and living conditions were collected using a questionnaire we developed. Information on medical and drug history and the type of dialysis (if any) was also collected. 

A Chinese version of the 14-question hospital anxiety and depression scale (HADS) was to assess anxiety and depression. The questionnaire has seven questions on depression and seven on anxiety. No anxiety/depression was defined as a score of 7 or less. A score of 8–10 indicated suspected anxiety/depression, and a score of 11 or higher indicated confirmed anxiety/depression [7]. To assess self-efficacy, we used a Chinese version of the chronic kidney disease self-efficacy instrument (CKD-SE), which consists of 25 questions [11]. Every question was scored from 0 to 10, resulting in a total score of 0–250, which indicated the level of confidence the patient possessed. For the self-management analysis, we used a 29-question Chinese version of the chronic kidney disease self-management instrument (CKD-SM) [19]. Each question was scored between 1 and 4, so the total score ranged from 29 to 116. Higher self-management and self-efficacy scores represented better management of CKD and a higher confidence in their own ability to deal with the disease.

### 2.4. Validity and Reliability

The content validity index (CVI) of the HADS was 0.9. The HADS was previously used in a study of anxiety and depression in patients post liver transplantation, yielding an internal consistency and reliability of 0.84 and a Cronbach’s α of 0.76 and 0.73 for anxiety and depression, respectively [20]. A Taiwanese study used a Mandarin Chinese version of the HADS to assess subjects undergoing haemodialysis, yielding Cronbach’s α values of 0.96 and 0.95 for the anxiety and depression subscales, respectively, and presented excellent internal consistency and reliability [21]. In the present study, when we used the HADS to measure the anxiety and depression levels of patients with CKD, we obtained Cronbach’s α values for these two categories of 0.91 and 0.93, respectively. The CVI of the CKD-SE and the CKD-SM were 0.96 and 0.95. The CKD-SE had previously been used on Taiwanese research subjects with CKD. Cronbach’s α for the scale was 0.91, indicating excellent reliability [22]. 

### 2.5. Data Analysis

For the Spearman rank correlation analysis, depression and anxiety levels were set at three levels: Low (scores ≤ 7), intermediate (scores of 8–10), and high (scores > 10). Self-management and -efficacy levels were categorized based on the first, second, and third quartiles of these indices (51.5, 62, and 71.5 for self-management and 67.5, 110.5, and 156.5 for self-efficacy). “High” self-management and -efficacy was defined as the upper quartile of each measure, and the lower three quartiles were defined as “low”. For the logistic regression analysis, depression was defined as a score of > 7 and education was considered high (value = 1) if the patient had completed at least senior high school. To evaluate the power of the current study, the correlation between the significant correlations was tested using fixed-scenario elements, setting alpha to 0.05.

### 2.6. Ethics Considerations

The present study was reviewed and approved by the institutional review board of the hospital (IRB no. T-2011-12-003). All subjects participated in the study voluntarily. After the researcher had explained the details of the study, participants signed an informed consent form and formally joined the study. Prior to completing the questionnaires, the participants were provided with the relevant information and told that they could opt out of the study without consequence or harm. To ensure privacy, anonymizing coding was used during the questionnaire completion, compilation, and data analysis. Data collected in the questionnaires were used solely for the purposes of academic research.

## 3. Results

This study included 112 patients, 69 were male (61.6%) and 43 were female (38.4%. We categorized 28 patients (25%) as having high self-management, 28 patients (25%) as having high self-efficacy, 84 patients (75%) as having low self-management, and 84 patients (75%) as having low self-efficacy (Table 1). The demographic characteristics and medical and treatment information for the participants are presented in Table 2. The distributions of the study variables according to the two classes of self-efficacy and self-management are given in Table 3 and Table 4.

### 3.1. Primary Outcomes

We performed a Spearman’s correlation analysis between the selected study variables (Table 5).

We performed a frequency analysis between the levels of anxiety and depression and the levels of self-management and self-efficacy (Table 6).

Using Spearman’s correlations, based on the depression and self-management measures, the power of the study was 82.1%, while based on the depression and self-efficacy measures, the power of the study was 86.2%. The generalized linear models for self-efficacy and self-management are given in Table 7.

### 3.2. Secondary Outcomes

To evaluate the impact of education and depression level on self-efficacy and self-management, we performed a logistic regression analysis and obtained the odds ratios and confidence intervals (Table 8). 

## 4. Discussion

We obtained the following findings. (1) The patients in the high self-management and self-efficacy categories were younger, had experienced a longer disease duration (since their diagnoses), and had a higher education level. (2) Self-management and self-efficacy were highly and significantly correlated with each other, and both were significantly correlated with depression. (3) Younger age, longer disease duration, and higher education levels were positively and independently associated with higher levels of self-management and self-efficacy, while having depression was negatively and independently associated with higher levels of self-management and self-efficacy. (4) Having depression decreased the odds of having high self-management and self-efficacy by 75.4% and 75.1%, respectively. (5) Being educated to a senior-high-school level or above increased the odds of having high self-management and self-efficacy by odds ratios of 4.47 and 3.56, respectively.

Younger patients have the confidence to cope with their illness, although they may still have illness-related fears [23]. They are also more adept at obtaining knowledge related to self-care, disease management, and disease control than older patients [24]. In addition, age is significantly related to problem-solving, with problem-solving ability decreasing in old age [3]. Sufficient knowledge about the disease and the ability to apply problem-solving and information-searching strategies is vital, and it is more likely that younger patients will seek help and information online, in discussions with professionals, and by reading scientific papers [3,25]. Younger patients are more proactive in seeking information from a range of sources, which enables them to effectively communicate on their situation, condition, and illness with health professionals [25]. Communication and collaboration between patients and health professionals is crucial for self-management [25,26,27]. 

The socioemotional selectivity theory suggests that the perception of time plays an important role in the pursuit of social goals such as self-management and self-efficacy. Social goals can be categorized as related to the acquisition of knowledge or the regulation of emotion. This theory suggests that if time is felt to be open-ended, knowledge-related goals are prioritized, but if time is perceived as limited, emotion-related goals are prioritized [28]. As time is likely to seem more limited to ESRD patients, the emotion-related goals may be more prominent. A younger patient with a high education level is likely to be able to search for information on the internet and from other publicly accessible sources [3]. A higher education level is usually associated with greater knowledge. Knowledge in general has a major impact on self-management, self-efficacy, and the foundations of decision making [3,10,29]. Illness-specific knowledge is also significantly positively correlated with self-management and self-efficacy [29].

This study indicates that self-management and self-efficacy are highly and significantly correlated with each other. This finding agrees with previous studies demonstrating that self-efficacy has a significant and reciprocal influence on overall self-management [3,7,11,19,25,29,30,31]. Patients with a high level of self-efficacy have better self-management, especially in the category of self-care [3,10]. Self-efficacy can help the patient to engage in self-management behaviours and vice versa [3,7,25]. 

Patients undergoing haemodialysis have a high prevalence of anxiety and depression [8,32], which disrupt their ability to address their own needs and reduce their attention levels [3]. Depression is a low-mood state that can affect a person’s thoughts, behaviour, and feelings, such that they may lose interest in activities and have problems communicating, concentrating, remembering, or making decisions [4,8,33]. Anxiety is a psychosocial and physiological state characterized by negative effects, both physical and emotional, on mental state and behaviour [3,4]. Mental-health conditions such as depression and anxiety have an important bearing on self-management and self-efficacy [4,17]. Our study shows that in pre-ESRD patients, self-management and self-efficacy are significantly correlated with depression but not with anxiety. These results contrast with other studies indicating that self-management and self-efficacy together can improve patients’ health since they enable them to perform tasks related to medical, role, and emotional management [8,32,34]. However, few studies have investigated the associations between these parameters. Studies with larger sample sizes would be able to confirm whether anxiety is an independent determinator of self-management and self-efficacy in pre-ESRD patients.

A study by Tsay et al. [35] evaluated the effects of an empowerment program for ESRD patients, which included the identification of problem areas for self-management, and determined that the program was effective for improving patients’ empowerment level, self-care, self-efficacy, and depression score. Our study suggests that the education level is also an important factor influencing self-efficacy and self-management in ESRD patients, which supports the idea that educational and empowerment programs can help ESRD patients increase their self-efficacy and self-management.

This study has several clinical implications. Special medical attention should be given to older, newly diagnosed ESRD patients with low education levels. Depressed patients should also be the focus of attention, since self-management and self-efficacy are highly and significantly correlated with depression.

A strength of this study was the power to determine the association between levels of depression and self-management or self-efficacy in pre-ESRD patients. A limitation was that it did not distinguish between the various components of self-management and self-efficacy in the analysis. Further studies on the relationship between depression and the components of self-management and self-efficacy are therefore recommended. Moreover, due to the cross-sectional nature of our study, it was not possible to infer causality in the relationships between depression, education, self-management, self-efficacy in pre-ESRD patients. Longitudinal studies would be required to investigate this.

## 5. Conclusions

We found that pre-ESRD patients with high levels of self-management and self-efficacy tended to be younger and have higher education levels. Depression and anxiety independently influenced the level of self-management and self-efficacy in pre-ESRD patients. The management and treatment of depression could benefit these patients. Increasing their level of education may also potentially increase their self-management and self-efficacy.

## Figures and Tables

**Table 1:** Distribution of high self-management and -efficacy in the study participants.

		Self-Efficacy		Total
		High, *n* (%)	Low, *n* (%)	
Self-Management	High	22 (78.57)	6 (7.14)	28 (25)
	Low	6 (21.43)	78 (92.86)	84 (75)
Total		28 (25)	84 (75)	112

**Table 2:** Demographic characteristics of the study participants.

Variable		Mean ± SD, *n* (%)
Age		70.16 ± 11.59
Years since diagnosis		4.28 ± 5.60
CKD stage	1	45 (41.28)
	2	37 (33.94)
	3	27 (24.77)
Sex (male)		69 (61.61)
Education	Illiterate	19 (16.96)
	Primary school	40 (35.71)
	Junior high school	21 (18.75)
	Senior high school	24 (21.43)
	College/University	8 (7.14)
Religion	Buddhism	20 (17.86)
	Taoism	86 (76.79)
	Other	6 (5.36)
Marital Status	Single	6 (5.36)
	Married	84 (75)
	Divorced	5 (4.46)
	Widowed	17 (15.18)
Employed (yes)		23 (20.54)
Living alone (yes)		11 (9.82)
Medical and drug history	HTN	93 (83.04)
	High FBS	65 (58.04)
	High LDL-C	19 (16.96)
	High cholesterol	41 (36.61)
	High uric acid	44 (39.29)
	Proteinuria	67 (59.82)
	High TRG	40 (35.71)
	Cancer (yes)	13 (11.61)
	Traditional medicine (yes)	12 (10.71)
Dialysis type	HD	26 (23.21)
	PD	4 (3.57)
	Hospice	17 (15.18)
	Other	69 (61.61)

CKD: Chronic kidney disease; FBS: Fasting blood sugar; HD: Haemodialysis; HTN: Hypertension; LDL: Low-density lipoprotein; PD: Peritoneal dialysis; TRG: Triglycerides.

**Table 3:** Distribution of variables according to the self-management level of the study participants.

Variable		Self-Management		
		Low, *n* (%)	High, *n* (%)	*p*
Age		72.72 ± 10.08	62.46 ± 12.58	<0.01
Years since diagnosis		3.30 ± 3.33	7.23 ± 9.12	<0.01
CKD stage	1	33 (73.33)	12 (2.67)	0.17
	2	31 (83.78)	6 (16.22)	
	3	17 (62.96)	10 (37.04)	
Sex (male)		55 (79.71)	14 (20.29)	0.17
Education	Illiterate	17 (89.47)	2 (10.53)	0.01
	Primary school	33 (82.50)	7 (17.5)	
	Junior high school	17 (80.95)	4 (19.05)	
	Senior high school	14 (58.33)	10 (41.67)	
	College/University	3 (37.50)	5 (62.5)	
Religion	Buddhism	13 (65)	7 (35)	0.39
	Taoism	67 (77.91)	19 (22.09)	
	Other	4 (66.67)	2 (33.33)	
Marital Status	Single	3 (50)	3 (50)	0.34
	Married	63 (75)	21 (25)	
	Divorced	5 (100)	0 (0)	
	Widowed	13 (76.47)	4 (23.53)	
Employed (yes)		14 (60.87)	9 (39.13)	0.10
Living alone (yes)		9 (81.82)	2 (18.18)	0.72
Medical and drug history	HTN	72 (77.42)	21 (22.58)	0.24
	High FBS	51 (78.46)	14 (21.54)	0.37
	High LDL-C	13 (68.42)	6 (31.58)	0.56
	High cholesterol	29 (70.73)	12 (29.27)	0.49
	High uric acid	34 (77.27)	10 (22.73)	0.82
	Proteinuria	48 (71.64)	19 (28.36)	0.37
	High TRG	32 (80)	8 (20)	0.49
	Cancer (yes)	7 (53.85)	6 (46.15)	0.08
	Traditional medicine (yes)	6 (50)	6 (50)	0.07
Dialysis type	HD	17 (65.38)	9 (34.62)	0.2
	PD	1 (25)	3 (75)	0.04
	Hospice	15 (88.24)	2 (11.76)	0.23
	Other	53 (76.81)	16 (23.19)	0.65

CKD: Chronic kidney disease; FBS: Fasting blood sugar; HD: Haemodialysis; HTN: Hypertension; LDL: Low-density lipoprotein; PD: Peritoneal dialysis; TRG: Triglycerides. Age is displayed in mean age (years) ± standard deviation.

**Table 4:** Distribution of variables according to the self-efficacy level of the study participants.

Variable		Self-Efficacy		
		Low, *n* (%)	High, *n* (%)	*p*
Age		73.20 ± 9.43	61.03 ± 12.82	<0.01
Years since diagnosis		3.23 ± 3.24	7.44 ± 9.11	<0.01
CKD stage	1	34 (75.56)	11 (24.4)	0.26
	2	31 (83.78)	6 (16.22)	
	3	18 (66.67)	9 (33.33)	
Sex (male)		52 (75.36)	17 (24.64)	1.00
Education	Illiterate	19 (100)	0 (0)	<0.01
	Primary school	31 (77.5)	9 (22.5)	
	Junior high school	16 (76.19)	5 (23.81)	
	Senior high school	14 (58.33)	10 (41.67)	
	College/University	4 (50)	4 (50)	
Religion	Buddhism	13 (65)	7 (35)	0.39
	Taoism	67 (77.91)	19 (22.09)	
	Other	4 (66.67)	2 (33.33)	
Marital Status	Single	3 (50)	3 (50)	0.47
	Married	63 (75)	21 (25)	
	Divorced	4 (80)	1 (20)	
	Widowed	14 (82.35)	3 (17.65)	
Employed (yes)		13 (56.52)	10 (43.48)	0.03
Living alone (yes)		9 (81.82)	2 (18.18)	0.72
Medical and drug history	HTN	74 (79.57)	19 (20.43)	0.02
	High FBS	54 (83.08)	11 (16.92)	0.02
	High LDL-C	14 (73.68)	5 (26.32)	1.00
	High cholesterol	29 (70.73)	12 (29.27)	0.49
	High uric acid	34 (77.27)	10 (22.73)	0.82
	Proteinuria	47 (70.15)	20 (29.85)	0.18
	High TRG	31 (77.5)	9 (22.5)	0.82
	Cancer (yes)	9 (69.23)	4 (30.77)	0.73
	Traditional medicine (yes)	7 (58.33)	5 (41.67)	0.17
Dialysis type	HD	15 (57.69)	11 (42.31)	0.03
	PD	1 (25)	3 (75)	0.04
	Hospice	16 (94.12)	1 (5.88)	0.99
	Other	54 (78.26)	15 (21.74)	0.37

CKD: Chronic kidney disease; FBS: Fasting blood sugar; HD: Haemodialysis; HTN: Hypertension; LDL: Low-density lipoprotein; PD: Peritoneal dialysis; TRG: Triglycerides. Age is displayed in mean age (years) ± standard deviation.

**Table 5:** Spearman’s rank correlation analysis between the selected study variables.

Variable		Education	CKD Stage	Depression	Anxiety	SE	SM
Education	*r*	1	-	-	-	-	-
	*p*	-	-	-	-	-	-
CKD stage	*r*	−0.15	1	-	-	-	-
	*p*	0.11	-	-	-	-	-
Depression	*r*	−0.09	0.09	1	-	-	-
	*p*	0.32	0.34	-	-	-	-
Anxiety	*r*	−0.02	0.08	0.51	1	-	-
	*p*	0.82	0.36	**<0.0001**	-	-	-
SE	*r*	0.28	0.07	−0.28	0.02	1	-
	*p*	**<0.01**	0.45	**<0.01**	0.81	-	-
SM	*r*	0.29	0.06	−0.26	−0.02	0.83	1
	*p*	**<0.01**	0.5	**<0.01**	0.75	**<0.0001**	-

CKD: Chronic kidney disease; SE: Self-efficacy; SM: Self-management; Bold: *p* < 0.05.

**Table 6:** Frequency analysis between three levels of anxiety and depression and four levels of self-management and self-efficacy.

Title		Depression			Anxiety		
		Low	Int.	High	Low	Int.	High
Self-Efficacy	Very low	14 (17.28)	6 (35.29)	8 (57.14)	23 (25)	3 (30)	2 (20)
	Low	22 (27.16)	5 (29.41)	1 (7.14)	25 (27.17)	1 (10)	2 (20)
	Int.	20 (24.69)	4 (23.53)	4 (28.57)	20 (21.74)	4 (20)	4 (40)
	High	25 (30.86)	2 (11.76)	1 (7.14)	24 (26.09)	2 (40)	2 (20)
	*p* ^†^	**0.02**			0.74		
Self-Management	Very low	16 (19.75)	7 (41.18)	5 (35.71)	22 (23.91)	3 (30)	3 (30)
	Low	18 (22.22)	4 (23.53)	5 (35.71)	23 (25)	1 (10)	3 (30)
	Int.	22 (27.16)	3 (17.65)	4 (28.57)	24 (26.09)	3 (30)	2 (20)
	High	25 (30.86)	3 (17.65)	0	23 (25)	3 (30)	2 (20)
	*p* ^†^	0.08			0.95		

^†^ Fisher’s exact test. Int.: Intermediate; Bold: *p* < 0.05.

**Table 7:** Generalized linear models for self-efficacy and self-management.

Variable		Self-Efficacy			Self-Management		
		Mean Square	*F*	*p*	Mean Square	*F*	*p*
Anxiety		3684.92	1.38	0.24	2.85	0.01	0.90
Depression		51,326.42	19.23	**<0.0001**	2528.79	12.73	**0.00**
Age		43,661.76	16.35	**0.00**	2035.44	10.25	**0.00**
Years since diagnosis		27,115.50	10.16	**0.00**	1491.98	7.51	**0.01**
CKD stage		569.87	0.21	0.65	0.13	0.00	0.98
Gender		3490.57	1.31	0.26	239.17	1.20	0.28
Education		13,760.53	5.15	0.03	1394.42	7.02	0.01
Religion		8650.54	3.24	0.08	360.29	1.81	0.18
Marital status		7.47	0.00	0.96	190.65	0.96	0.33
Employed (yes)		291.67	0.11	0.74	13.37	0.07	0.80
Living alone (yes)		251.86	0.09	0.76	149.86	0.75	0.39
Medical and drug history	HTN	420.92	0.16	0.69	12.51	0.06	0.80
	High FBS	1359.91	0.51	0.48	111.24	0.56	0.46
	High LDL-C	210.82	0.08	0.78	50.95	0.26	0.61
	High cholesterol	857.04	0.32	0.57	24.77	0.12	0.72
	High uric acid	3490.13	1.31	0.26	247.55	1.25	0.27
	Proteinuria	1783.15	0.67	0.42	124.66	0.63	0.43
	High TRG	74.23	0.03	0.87	31.88	0.16	0.69
	Cancer (yes)	3669.15	1.37	0.24	78.37	0.39	0.53
	Traditional medicine (yes)	916.73	0.34	0.56	54.29	0.27	0.60
Dialysis type	HD	1783.23	0.67	0.42	4.16	0.02	0.89
	PD	1318.39	0.49	0.48	262.65	1.32	0.25
	Hospice	1290.90	0.48	0.49	25.03	0.13	0.72
	Other	715.48	0.27	0.61	4.62	0.02	0.88

CKD: Chronic kidney disease; FBS: Fasting blood sugar; HD: Haemodialysis; HTN: Hypertension; LDL: Low-density lipoprotein; PD: Peritoneal dialysis; TRG: Triglycerides; Bold: *p* < 0.05.

**Table 8:** Logistic regression analysis between depression and education levels and self-efficacy and self-management.

	Self-Management			Self-Efficacy		
	OR	CI	*p*	OR	CI	*p*
Depression	0.246	0.06–0.92	**0.03**	0.249	0.06–0.92	0.03
Education	4.47	1.74–11.45	**<0.001**	3.56	1.40–9.03	<0.001

CI: Confidence interval; OR: Odds ratio; Bold: *p* < 0.05.

## Data Availability

The data presented in this study are available on request from the corresponding author. The data are not publicly available due to institutional review board statement of Cardinal Tien Hospital.

## References

[1] Carney E.F. (2020). The impact of chronic kidney disease on global health. Nat. Rev. Nephrol..

[2] Tsai M.H., Hsu C.Y., Lin M.Y., Yen M.F., Chen H.H., Chiu Y.H., Hwang S.J. (2018). Incidence, Prevalence, and Duration of Chronic Kidney Disease in Taiwan: Results from a Community-Based Screening Program of 106,094 Individuals. Nephron.

[3] Li H., Jiang Y.F., Lin C.C. (2014). Factors associated with self-management by people undergoing hemodialysis: A descriptive study. Int. J. Nurs. Stud..

[4] Goh Z.S., Griva K. (2018). Anxiety and depression in patients with end-stage renal disease: Impact and management challenges—A narrative review. Int. J. Nephrol. Renov. Dis..

[5] Bradbury B.D., Fissell R.B., Albert J.M., Anthony M.S., Critchlow C.W., Pisoni R.L., Port F.K., Gillespie B.W. (2007). Predictors of early mortality among incident US hemodialysis patients in the Dialysis Outcomes and Practice Patterns Study (DOPPS). Clin. J. Am. Soc. Nephrol. CJASN.

[6] Curtin R.B., Mapes D.L. (2001). Health care management strategies of long-term dialysis survivors. Nephrol. Nurs. J. J. Am. Nephrol. Nurses’ Assoc..

[7] Wu S.F.V., Lee M.C., Hsieh N.C., Lu K.C., Tseng H.L., Lin L.J. (2018). Effectiveness of an innovative self-management intervention on the physiology, psychology, and management of patients with pre-end-stage renal disease in Taiwan: A randomized, controlled trial. Jpn. J. Nurs. Sci..

[8] Lee M.C., Wu S.V., Hsieh N.C., Tsai J.M. (2016). Self-Management Programs on egfr, Depression, and Quality of Life among Patients with Chronic Kidney Disease: A Meta-Analysis. Asian Nurs. Res..

[9] Griva K., Mooppil N., Seet P., Krishnan D.S.P., James H., Newman S.P. (2011). The NKF-NUS hemodialysis trial protocol—A randomized controlled trial to determine the effectiveness of a self management intervention for hemodialysis patients. BMC Nephrol..

[10] Gela D., Mengistu D. (2018). Self-management and associated factors among patients with end-stage renal disease undergoing hemodialysis at health facilities in Addis Ababa, Ethiopia. Int. J. Nephrol. Renov. Dis..

[11] Lin C.C., Wu C.C., Anderson R.M., Chang C.S., Chang S.C., Hwang S.J., Chen H.C. (2012). The chronic kidney disease self-efficacy (CKD-SE) instrument: Development and psychometric evaluation. Nephrol. Dial. Transplant..

[12] Bandura A. (1978). Self-efficacy: Toward a unifying theory of behavioral change. Adv. Behav. Res. Ther..

[13] Bandura A., Cervone D. (1983). Self-evaluative and self-efficacy mechanisms governing the motivational effects of goal systems. J. Personal. Soc. Psychol..

[14] Bandura A. (1997). Self-Efficacy: The Exercise of Control.

[15] Guide for Constructing Self-Efficacy Scales. https://www.uky.edu/~eushe2/Bandura/BanduraGuide2006.pdf.

[16] Shokri S., Akbari B. (2016). Relationship of self-efficacy with life expectancy and death. Electron. J. Biol..

[17] Tahmassian K., Moghadam N.J. (2011). Relationship between self-efficacy and symptoms of anxiety, depression, worry and social avoidance in a normal sample of students. Iran. J. Psychiatry Behav. Sci..

[18] Tsay S.L., Healstead M. (2002). Self-care self-efficacy, depression, and quality of life among patients receiving hemodialysis in Taiwan. Int. J. Nurs. Stud..

[19] Lin C.C., Wu C.C., Wu L.M., Chen H.M., Chang S.C. (2013). Psychometric evaluation of a new instrument to measure disease self-management of the early stage chronic kidney disease patients. J. Clin. Nurs..

[20] Pelgur H., Atak N., Kose K. (2009). Anxiety and depression levels of patients undergoing liver transplantation and their need for training. Transplant. Proc..

[21] Chen P., Kuo S., Chang H., Liu Y., Hsu T.J. (2007). Preliminary study of the relationship between health locus of control, psychological distress and health promotion behavior in a group of hemodialysis patients. J. Taiwan Nephrology Nurses Association.

[22] Lee M.C., Lu K.C., Wu S.F.V., Hsieh H.L., Liu Y.M. (2016). Effectiveness of Self-Management Program on Improving Self-Efficacy and Depression in Patients with Hemodialysis. Taipei City Med. J..

[23] Leventhal E.A. (1984). Aging and the perception of illness. Res. Aging.

[24] Kim S., Kim E., Ryu E. (2019). Illness Perceptions, Self-Care Management, and Clinical Outcomes According to Age-Group in Korean Hemodialysis Patients. Int. J. Environ. Res. Public Health.

[25] Curtin R.B., Walters B.A., Schatell D., Pennell P., Wise M., Klicko K. (2008). Self-efficacy and self-management behaviors in patients with chronic kidney disease. Adv. Chronic Kidney Dis..

[26] Welch J.L., Johnson M., Zimmerman L., Russell C.L., Perkins S.M., Decker B.S. (2015). Self-management interventions in stages 1 to 4 chronic kidney disease: An integrative review. West. J. Nurs. Res..

[27] Nunes J.A.W., Wallston K.A., Eden S.K., Shintani A.K., Ikizler T.A., Cavanaugh K.L. (2011). Associations among perceived and objective disease knowledge and satisfaction with physician communication in patients with chronic kidney disease. Kidney Int..

[28] Carstensen L.L., Isaacowitz D.M., Charles S.T. (1999). Taking time seriously: A theory of socioemotional selectivity. Am. Psychol..

[29] Hafezieh A., Dehghan M., Taebi M., Iranmanesh S. (2020). Self-management, self-efficacy and knowledge among patients under haemodialysis: A case in Iran. J. Res. Nurs..

[30] Fan J.-L., Kong Y., Shi S.-H., Cheng Y.-H. (2016). Positive correlations between the health locus of control and self-management behaviors in hemodialysis patients in Xiamen. Int. J. Nurs. Sci..

[31] Lin C.-C., Tsai F.-M., Lin H.-S., Hwang S.-J., Chen H.-C. (2013). Effects of a self-management program on patients with early-stage chronic kidney disease: A pilot study. Appl. Nurs. Res..

[32] Lin M.-Y., Liu M.F., Hsu L.-F., Tsai P.-S. (2017). Effects of self-management on chronic kidney disease: A meta-analysis. Int. J. Nurs. Stud..

[33] Vork D.L., Schneekloth T.D., Bartley A.C., Vaughan L.E., Lapid M.I., Jowsey-Gregoire S.G., El-Zoghby Z.M., Herrmann S.M., Tran C.L., Albright R.C. (2018). Younger Adults Initiating Hemodialysis: Antidepressant Use for Depression Associated With Higher Health Care Utilization. Proceedings of Mayo Clinic Proceedings.

[34] Kokoszka A., Leszczyńska K., Radzio R., Daniewska D., Łukasiewicz A., Orzechowski W.M., Piskorz A., Gellert R. (2016). Prevalence of depressive and anxiety disorders in dialysis patients with chronic kidney disease. Arch Psychiatry Psychother.

[35] Tsay S.L., Hung L.O. (2004). Empowerment of patients with end-stage renal disease—A randomized controlled trial. Int. J. Nurs. Stud..

